# Nitrous Oxide Inhalant Abuse: Preliminary Results from a Cross-Sectional Study on Knowledge, Attitudes, and Practices of Italian Physicians (2023)

**DOI:** 10.3390/medicina59101820

**Published:** 2023-10-12

**Authors:** Matteo Riccò, Pietro Ferraro, Silvia Corrado, Marco Bottazzoli, Federico Marchesi

**Affiliations:** 1 Occupational Health and Safety Service on the Workplace/Servizio di Prevenzione e Sicurezza Ambienti di Lavoro (SPSAL), Department of Public Health, AUSL–IRCCS di Reggio Emilia, 42122 Reggio Emilia, Italy; 2 Occupational Medicine Unit, Direzione Sanità, Italian Railways’ Infrastructure Division, RFI SpA, 00161 Rome, Italy; dott.pietro.ferraro@gmail.com; 3 ASST Rhodense, Dipartimento della donna e Area Materno-Infantile, UOC Pediatria, 20024 Garbagnate Milanese, Italy; scorrado@asst-rhodense.it; 4 Department of Otorhinolaryngology, APSS Trento, 31223 Trento, Italy; marco.bottazzoli@apss.tn.it; 5 Department of Medicine and Surgery, University of Parma, 43126 Parma, Italy; federico.marchesi@unipr.it

**Keywords:** nitrous oxide, toxicology, vitamin B12, knowledge, attitudes, practice

## Abstract

*Background and Objectives*: Nitrous oxide (N_2_O) has recently emerged as a cheap alternative to other recreational substances. Although legally available, its chronic use is associated with severe neurological and hematological complications due to the irreversible inactivation of vitamin B12. While no reliable data on abuse of N_2_O in Italy have been provided to date, we assessed the knowledge, attitudes, and practices of Italian medical professionals on the management of N_2_O abuse cases. *Materials and Methods*: A cross-sectional study was performed as a web-based survey through a series of Facebook discussion groups (targeted medical professionals: 12,103), and participants were specifically asked about their previous understanding of N_2_O abuse and whether they had or not any previous experience in this topic. *Results*: A total 396 medical professionals participated in the survey. Overall, 115 participants had previous knowledge about N_2_O abuse (29.04%), with higher odds for professionals with a background in emergency medicine (adjusted odds ratio (aOR) 3.075; 95% confidence intervals (95%CI) 1.071 to 8.828) and lower for specialists in psychiatry (aOR 0.328; 95%CI 0.130 to 0.825). Knowledge status on N_2_O abuse was largely unsatisfying, as knowledge status, reported as a percent value, was estimated to 45.33% ± 24.71. Having previously managed a case of N_2_O abuse was associated with higher risk perception of the actual severity of this condition (aOR 5.070; 95%CI 1.520 to 16.980). *Conclusions*: Our study suggests that N_2_O poisoning cases are occurring in Italian settings but are not reasonably reported to national authorities. As substantial knowledge gaps of Italian medical workforces were identified, we cannot rule out that the actual abuse of N_2_O in the population may be far larger than currently suspected.

## 1. Introduction

Nitrous oxide (dinitrogen oxide, N_2_O; CAS number 10024-97-2) is a non-combustible, odorless, and colorless gas with various legitimate medical, industrial, commercial, and scientific uses [1,2,3,4,5,6]. The World Health Organization (WHO) has included N_2_O in its list of essential medicines due its anesthetic and pain-reducing effect [7]. Of interest, N_2_O is also known as “laughing gas” as a result of the transient feeling of detachment, euphoria, relaxation, and calmness that it causes when inhaled [1,2]. The recreational use of N_2_O dates back to 1772, when “laughing gas parties” were originally reported by Humphry Davy [1,2,6]. After decades of limited use as a recreational substance due to the high costs and the difficulty to obtain it in anything other than large tanks, by the early 1970s, N_2_O had re-emerged as a legal, widely available, and cheap alternative to other substances [8]. To date, N_2_O is legally and commercially available in small, pressurized metal cartridges containing up to 8 g of N_2_O (equal to around 4 L of gas), originally designed for the food industry. Typically, the user inhales the gas by discharging the cartridges into an empty balloon or directly into the mouth and exhales it either into the air or rebreathes it for extra effect. Smaller, pocket-sized cartridges (known as “crackers”) are frequently used in a similar way: Interestingly, when the cracker is opened, and the gas is released into the balloon, the metal cartridge freezes (−40 °C to −55 °C), causing cold burns to the hands when directly exposed. Moreover, some users inhale the N_2_O from cylinders or the cartridges through a tube or a face mask or directly from the dispenser. While the former option poses a substantial risk of accidental asphyxiation, the latter is strongly associated with the risk of cold burns or airways injuries [6,8,9,10]. Whether N_2_O can or cannot cause dependence still remains uncertain, but there is considerable evidence that the large majority of N_2_O users does not develop problematic use [1,2,11], only consuming small quantities occasionally, a few times a year [8].

N_2_O causes the functional inhibition of vitamin B12 or cobalamin [1,2,3,5,12,13,14]. Vitamin B12 is an important coenzyme for methionine synthase, the enzyme that catalyzes the transmethylation of homocysteine into methionine, which in turn is essential in both DNA and myelin synthesis [3,14,15,16,17,18,19]. As recently summarized by Marsden et al. [1], chronic vitamin B12 deficiency elicited by N_2_O abuse therefore results in macrocytic anemia, accumulation of homocysteine, and neurotoxicity, with variable degree of demyelination of the spinal cord (mostly in its cervical section), peripheral nerves, and white matter of the brain [1,2], which in turn causes the most common cause of medical presentation, i.e., sensory changes, gait disturbances, or limb weakness [1,2,3,12]. As high levels of homocysteine represent a well-known pro-thrombotic risk factor, chronic use of N_2_O has unsurprisingly resulted in the increased occurrence of cerebrovascular and thromboembolic diseases, including deep vein thrombosis and pulmonary embolism [20,21,22,23]. Moreover, demyelination as well as the direct effect of N_2_O on N-methyl-D-aspartate (NMDA) receptors in the brain thrush the inhibition of excitatory glutamatergic neurotransmission via non-competitive inhibition and could result in psychiatric sequelae ranging from mood disorders to hallucinations and psychosis [14,15,24,25,26,27]. From a toxicological point of view, N_2_O abuse is characterized by some distinctive features. On the one hand, as the functional inhibition of vitamin B12 is dose-dependent, myeloneuropathy or anemic effects are rare despite the widespread recreational use of N_2_O [1]. Moreover, severe complications such as thrombotic sequelae usually occur, associated with neurological, hematological, and psychiatric signs and symptoms of chronic N_2_O abuse [1,2,4,5,12,13,20,28,29]. On the other hand, the prompt discontinuation of N_2_O and the supplementation of vitamin B12 rapidly improve the clinical features of the patients, potentially leading to the extensive resolution of signs and symptoms in the large majority of reported cases [1,2,3,14,30,31,32,33].

Since 2017–2018 [8], an increased notification of clinical cases reasonably associated chronic N_2_O abuse (i.e., the daily use of 100 × 8 g cartridges for at least one month) [1] has been reported from most high-income countries and mainland China [1,2,12,34]. As N_2_O can be legally acquired, and the large majority of its users does not develop any signs or symptoms, information about the prevalence of N_2_O use is generally limited and based on local registries and cross-sectional studies [1,8,9]. However, a general population survey from the Netherlands identified a 12-month prevalence rate of 14.5% among young adults (18–19 years) and 12.1% among adults ages 20 to 24 years, with alarming estimates from younger age groups (6.7% for subjects 15 years old and 11.7% for those 16 years old) [30]. Similar estimates were provided by a 2019 survey from United Kingdom (UK), as 8.7% of subjects aged 16 to 24 years admitted a lifetime prevalence [11]. Available surveys from Denmark (13.5% lifetime prevalence in people aged 15 to 25 years and 6.5% for the last 12 months) [8] and from France (5.5% lifetime use in a sample of students aged 14 to 15 years; lifetime use of 29% in a sample of 981 medical students from the Parisian area) have stressed that N_2_O abuse should be acknowledged as a common issue for the whole of the European Union/European Economic Area (EU/EEA) [8,35,36,37]. Although the first post-pandemic report about substances abuse in the UK suggests a slight reduction in the use of N_2_O as a recreational drug (3.9% in age group 16–24 years in 2021–2022 compared to 8.7–8.8% in 2017–2010) [11,38], laughing gas has been reported among the most frequently used drugs, following cannabis and cocaine.

To date, despite some episodic reports from the general press, the actual prevalence of N_2_O abuse in Italy remains largely unknown. Moreover, the data from European Monitoring Centre for Drugs and Drug Addictions (EMCDDA) and the European Drug Report 2023 have been unable to provide any estimate for both prevalence and pattern of N_2_O abuse in Italy [8]. Nevertheless, the lack of reported cases might imply an underestimation of the actual N_2_O (ab)use in Italy rather than a real lack of clinical cases. As the appropriate understanding about this substance of abuse and its effects is instrumental in achieving an appropriate and timely diagnosis and in improving the actual prognosis of the patients [1,9], the proper assessment of knowledge (i.e., what is actually known about a certain condition), attitudes (i.e., the set of emotions, beliefs, and behaviors toward a particular object, person, thing, or event), and practices (i.e., what is actually done in the context of the topic of interest; collectively KAP) of medical professionals on N_2_O abuse could provide an estimate of healthcare delivery, which is how the medical workforce is able to cope with incident cases and their requirements. While Italy is seemingly exempted from the growing concerns otherwise reported in other European Union countries [8], doubts on the real-world prevalence are arising. We therefore designed the present cross-sectional study in order to ascertain (a) whether Italian N_2_O abuse cases have actually occurred, (b) whether Italian medical professionals have received any formation on the management of resulting disorders, (c) their actual risk perception of N_2_O abuse, and (d) the main determinants of their risk perception.

## 2. Materials and Methods

### 2.1. Study Design

The present study was designed as a cross-sectional survey (see the strengthening the reporting of observational studies in epidemiology (STROBE) checklist as Appendix A Table A1) [39] and was performed between 22 August 2023 and 29 August 2023. In order to reduce turnaround time, we opted for a web-based delivery of the questionnaire through a series of five closed Facebook discussion groups. The aforementioned groups belonged to the Facebook Community “Memedical”, whose characteristics have been described elsewhere [40,41]. Briefly, the community was founded during the SARS-CoV-2 pandemic for providing mutual help between medical professionals from the whole of Italy [40]. This community was chosen for the delivery of the questionnaire because potential participants are required to share with administrators their medical board registration number, which is then double checked by means of the application provided by the Italian Federation of Medical Boards (FNOMCEO; https://portale.fnomceo.it/cerca-prof/index.php; accessed on 1 September 2023). Moreover, the aforementioned community not only encompasses a large number of professionals from the whole of Italy but also includes various specialties and subspecialties as well as medical settings (primary care, hospitals, etc.). At the time of the survey, the group had 12,103 unique members, but no information could be obtained regarding how many of them were active participants.

The authorization for sharing the study invitation through the discussion groups was granted by group administrators upon a specific request of chief researcher (M.R.), who posted the invitation to the survey, including the appropriate link to a specifically designed module of Google Forms (Google LLC; Menlo Park, CA, USA). The first page of the module included the full informed consent (Appendix A Table A2) and the consent for study participation through a mandatory, dichotomous question (i.e., yes vs. no). All participants agreeing with the informed consent were then asked whether or not they were a medical professional (yes vs. no). In both cases, a negative answer led to the end of questionnaire. Participants agreeing with the informed consent and having a medical background received a further dichotomous item asking whether or not they had any knowledge regarding N_2_O as a recreational drug. A positive answer led to the full questionnaire, while a negative one led to the demographic section, with no further options for reviewing the remaining items.

### 2.2. Sample Size Calculation

As no preventive estimates on the previous professional experience of the Italian medical workforce with N_2_O abuse were available at the time of the study, we assumed an a priori probability that half of the potential participants had any knowledge that N_2_O can be used as a recreational substance. As a consequence, assuming a Type I error of 5% (0.05) and power of 95%, the minimum sample size (N) was calculated as follows:

N = 1.96^2^ × 0.5 × (1 − 0.5)/0.05^2^ = 3.8416 × 0.5 × 0.5/0.0025 = 384 (1)

### 2.3. Questionnaire

The questionnaire was designed as an anonymous one. Therefore, personal data (e.g., name, IP address, and email address) and any personal information not strictly related to the survey, particularly clinical information, were not requested, saved, or tracked. Even when dealing with demographic data, such information was requested in generic terms. No monetary compensation was offered to the participants, but they were guaranteed that at the end of the questionnaire, a full explanation of all items would be provided, representing an educative opportunity on N_2_O.

As no specific questionnaire has been previously validated, the present instrument was specifically designed for this study, and its content followed the blueprint of previous questionnaires employed in KAP studies on medical professionals [42,43,44,45,46]. The test–retest reliability of the questionnaire was preventively assessed through a survey of 15 healthcare workers (HCWs). Testers received the beta testing version of the questionnaire via email and were asked to complete the questionnaire at two different points in time (T1 = 1 July 2023; T2 = 15 July 2023). The paired comparison of all items was performed by calculation of Spearman’s rank test correlation coefficient (rho). Items having a rho > 0.800 were considered “consistent” and were included in the final questionnaire that was then delivered by August 2023. Beta testing questionnaires were not included in this study. Items removed from the questionnaire are provided in Appendix A.1 and Appendix A.2.

The English translation of the final questionnaire is available as Appendix A.1 and Appendix A.2 and includes the following sections:

1. Main demographic data: age, gender, seniority, kind of medical background (i.e., general practitioner, emergency department, internal medicine, neurology, psychiatry, or other), and the Italian region where the professional mainly worked and lived;
2. Knowledge test: According to the medical applications of health belief model (HBM) [46,47,48,49,50], knowledge status of a certain professional about a specific topic is key determinant of attitudes and behaviors, which in this specific case leads to the appropriate management of N_2_O abuse cases. In order to ascertain the knowledge status of participants, they received a total of 20 statements about N_2_O, including 16 dichotomous items (e.g., “N_2_O irreversibly inactivates vitamin B12”; TRUE) and 4 polytomous ones (e.g., “Chronic abuse of N_2_O is frequently associated with magnetic resonance imaging (MRI) anomalies…” (a) of the cerebral cortex; (b) of the brainstem; (c) of the cervical spinal cord; or (d) of the thorax spinal cord; correct answer = c); the survey was therefore designed through an extensive review of the medical literature [1,2,3,12,13,20,28,30]. More precisely, most of the items were identified through the analysis of the recent report from EMCDDA [8]. In order to ascertain whether the items regarding the knowledge were able to properly discriminate between participants with “strong” and “weak” understanding of N_2_O abuse, the approach suggested by Möltner and Jünger was applied [51,52]. Briefly, the correlation of each item of the knowledge test with the sum of all corrected answers was assessed through Spearman’s rank test; all questions with a rho ≥ 0.4 were included in a summary score (knowledge score; GKS). In accord with the original model provided by the studies of Betsch and Wicker [46] and Zingg and Siegrist [45], in order to stress the appropriate answers over the inappropriate ones and the lack of knowledge over a specific topic, GKS was calculated by adding +1 to the sum score for every correct answer, whereas a wrong indication or a missing/“don’t know” answer added 0;
3. Risk perception: According to the original report from Yates [49], perceived risk can be defined by the perceived probability of a certain event (F) and the expected consequences of that event (C). Participants were therefore requested to rate the perceived severity (C^N2O^) and the perceived frequency (F^N2O^) of N_2_O recreational abuse in the Italian population by means of a fully labeled 5-point Likert scale (range: from “not significant” with a score of 1 to “very significant” with a score of 5). A cumulative risk perception score (RPS) was therefore calculated as follows:
  C^N2O^ × F^M2O^ = RPS (2)
  Respondents were then asked to rate how difficult they perceived the management of N_2_O abuse in Italian settings compared to other abuse substances, including cocaine, opioids, cannabinoids, and amphetamines. All of the aforementioned disorders were rated 1 (not difficult) to 10 (very difficult);
4. Attitudes: For the aims of the present study, the attitude was acknowledged as the tendency that is expressed by evaluating a particular entity with some degree of favor or disfavor [53]. Therefore, reporting a certain attitude involved the expression of an evaluative judgment about a certain item. Respondents were therefore requested to rate, through a full Likert scale from 1 (totally disagree) to 5 (totally agree), whether or not they perceived N_2_O abuse cases as a likely occurrence during daily activities in the following months. Through a subsequent item, they were asked whether or not they perceived N_2_O as potentially affecting daily working activities and whether or not they were confident of being able to recognize a N_2_O abuse case;
5. Practices: Participants were requested to report whether or not they had received any previous medical formation on N_2_O abuse and its management and whether they had previously managed any case of N_2_O abuse (ever vs. never).

### 2.4. Ethical Considerations

The present study was designed as opinion survey, and no personal information that would lead either directly or indirectly to the identification of participants was gathered. Moreover, no other data (i.e., email address, IP address, etc.) that could allow the identification of the participant were otherwise collected. The informed consent preventively guaranteed all participants about the anonymous design of the questionnaire as well as the confidentiality of all retrieved data and that all gathered information would be handled confidentially and only collectively analyzed. In order to avoid any potential detrimental consequence of the inappropriate understanding of the items included in the questionnaire (i.e., stress, anxiety, and even panic), only participants with at least a basic understanding of this topic were allowed to check the knowledge test, and the correct answers to all items were available as a plain text upon the completion of the questionnaire. In summary, the present study reasonably caused no stigma or harm to the participants. As the present study did not retrieve any clinical data from the participants, and no individual or personal information that could lead to the direct or indirect identification of the participants was included, it was not configured according to clinical trial law (Gazzetta Ufficiale no. 76, dated 31 March 2008) [54] (Supplementary File S1) but rather as an opinion survey. As the present study only processed anonymous information for statistical and research purposes, the preventive assessment by the competent Ethical Committee and Institutional Review Board was not statutorily required, according to Italian and European Regulation 2016/679, point n.26 [54,55].

### 2.5. Data Analysis

All continuous variables were reported as average ± standard deviation (SD), while categorical ones were reported as percent values. As a preliminary step, GKS and RPS were normalized as percent values that were then dichotomized by median value as high (>median) vs. low estimates (≤median). Likert scales were also dichotomized as follows: Values for “agree” and “totally agree” were aggregated as “somewhat agreeing”, while values ranging from “totally disagree” to “neutral” were aggregated as “somewhat disagreeing”.

Distribution of continuous variables was tested through the D’Agostino and Pearson K2 test. Normality distribution was rejected for all *p*-values < 0.10, and variables were therefore compared through Mann–Whitney or Kruskal–Wallis tests for multiple independent samples, while their correlation was assessed through calculation of the Spearman’s rank correlation coefficient. On the other hand, a *p*-value ≥ 0.10 identified a normal distribution, and the variables were compared through the Student’s *t*-test for unpaired data or ANOVA, where appropriate, and their association was assessed by means of Pearson’s correlation test.

Categorical variables were reported by the outcome variables of having or not any previous understanding of the N_2_O as an abuse substance and having or not previously managed a case of N_2_O abuse, whether chronic or acute. These were analyzed through chi-square test with continuity correction. Internal consistency of the knowledge sections and its reliability were measured through calculation of the Cronbach’s alpha. Cronbach’s alpha (also known as rho-equivalent reliability) is a measure of how closely related a set of items are as a group. Even though no universally accepted cut-off values do exist, a score ≥ 0.7 is considered for the acceptable reliability of the questionnaire.

A multivariable analysis was then performed through two distinctive models of binary logistic regression analysis, with calculation or adjusted odds ratio (aOR) and their respective 95%CI.

In model I, the outcome variable was represented by having or not any previous understanding of N_2_O as an abuse substance. In model II, the outcome variable was having or not previously managed any N_2_O abuse case. In both models, explanatory variables were all categorical variables that at univariate analysis were significantly associated (i.e., *p* < 0.05) with outcome variables. Statistical analyses were performed by means of IBM SPSS Statistics 26.0 for Macintosh (IBM Corp., Armonk, NY, USA), R (version 4.3.1) [19] and Rstudio (version 2023.06.0 Build 421; Rstudio, PBC; Boston, MA, USA) software by means of the packages epiR (version 2.0.62) and fmsb (version 0.7.5).

## 3. Results

### 3.1. Descriptive Analysis

**Figure 1:**
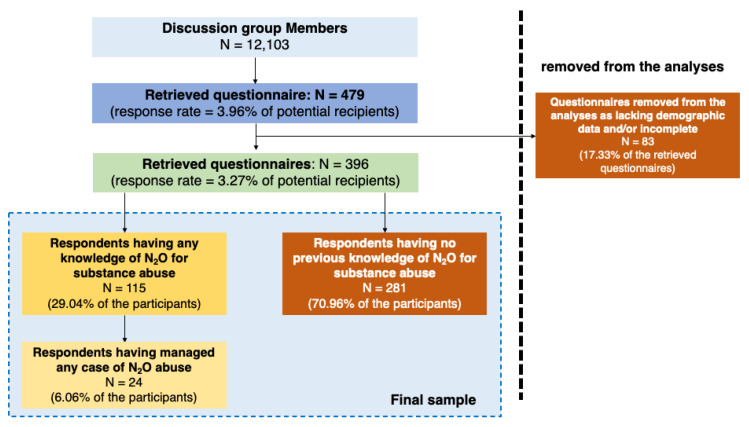
Flow chart of the selection of study participants.

As shown in Figure 1, a convenience sample of 479 medical professionals (3.96% of the potentially eligible population) completed the questionnaire and participated in the survey. However, 83 questionnaires (17.33% of the initial sample) lacked demographic data and/or were incomplete and were therefore removed from the analyses. The final sample eventually included a total of 396 questionnaire (response rate = 3.27% of potential recipients). Overall, 115 participants (29.04% of the final sample) had any previous knowledge of N_2_O as a substance of abuse, and 24 (6.06%) had any previous personal experience in the management of N_2_O abuse cases.

The demographic characteristics of the participants included in the final sample are reported in Table 1. The majority of them were of female gender (63.38%), and the mean age was 42.70 years ± 9.71, while their seniority was estimated to 16.10 years ± 9.47. Of them, 29.04% reported less than 20 years of total seniority as medical professionals. Around half of respondents lived in Northern Italy (49.75%), followed by Central Italy (29.04%), Southern Italy (13.64%), and the major islands of Sicily and Sardinia (7.58%). The most frequently reported information source was represented by formation courses belonging to medical continuing education programs (83.84%), followed by official websites from governmental bodies, scientific societies and health authorities (82.32%), other healthcare workers (56.82%), medical journals (36.11%), non-official websites (13.89%), and social media (10.86%). No one among respondents included friends and relatives as information sources.

### 3.2. Knowledge Test

**Figure 2:**
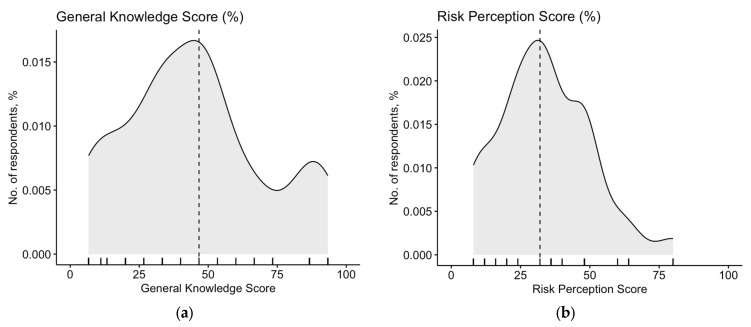
Density plots for: (**a**) knowledge score in 115 Italian physicians participating in the survey; (**b**) risk perception score (RPS). Both GKS (D’Agostino–Pearson’s normality test K2 = 5.747; *p*-value = 0.057) and RPS (K2 = 5.453; *p* value = 0.066) were somehow skewed and did not pass the normality check. Dotted line represents median value (46.67% and 32.0%, respectively).

A total of 115 participants (29.04% of final sample) had any knowledge of the abuse of N_2_O as a recreational substance and received the full knowledge test including 20 items. The individual answers and the correlation of each item with the corresponding cumulative score (potential range, 0 to 20; mean 9.44 ± 4.00; median 10; actual range 3 to 19) are reported in Appendix A Table A7. Briefly, items D1, D4, D5, D9, and D20 were associated with the cumulative score with a rho < 0.3 and were therefore removed from the analyses. GKS was therefore calculated over a total of 15 items. After percent normalization, an unsatisfying GKS estimate of (50.10% ± 22.51; actual range 6.67% to 94.44%, median 46.67%) was calculated (Figure 2a, Table 2), and its distribution did not pass the normality check (D’Agostino–Pearson normality test K = 5.747, *p* = 0.057). The internal consistency coefficient amounted to Cronbach’s alpha = 0.828, suggesting an acceptable reliability of the questionnaire.

### 3.3. Risk Perception

When dealing with the perceived frequency of N_2_O abuse in Italy, only 8.70% of the total participants rated this condition as frequent or very frequent. On the contrary, the majority of participants (53.04%) reportedly characterized N_2_O abuse as a potentially severe or very severe condition. As a consequence, the cumulative RPS was estimated to 33.67% ± 16.32 (actual range: 8.0% to 80.0%, median 32.0%), and its overall distribution was visually and statistically skewed (Figure 2b; D’Agostino–Pearson K2 = 5.453, *p* = 0.066).

### 3.4. Attitudes

In total, 16.52% of participants agreed or totally agreed that N_2_O abuse cases could become a daily occurrence in daily practice, while 12.17% responded that N_2_O abuse cases could potentially affect daily working activities (Table 2). Overall, only 9.56% of total respondents were confident of being able to properly recognize a N_2_O abuse case.

**Figure 3:**
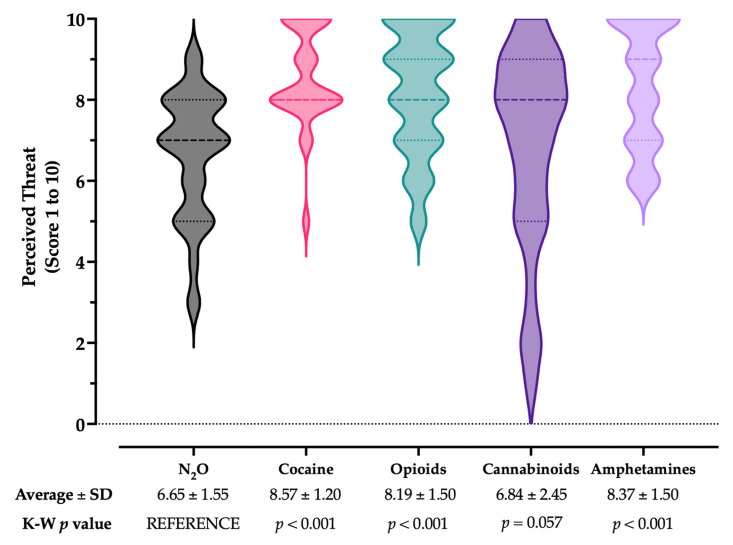
Box and violin plot for the perceived burden on National Health Service of N_2_O abuse compared to the abuse of cocaine, opioids, cannabinoids, and amphetamines. Comparisons were performed by mean of the Kruskal–Wallis (K–W) test for multiple comparisons by assuming N_2_O as the reference group.

When asked to rate the perceived threat associated with N_2_O abuse cases, with a potential range 0 to 10, a score of 6.65 ± 1.55 was reported compared to 8.57 ± 1.20 for cocaine, 8.19 ± 1.50 for opioids, 6.84 ± 2.45 for cannabinoids, and 8.37 ± 1.50 for amphetamines (Figure 3). Assuming N_2_O as the reference group, estimates for cocaine, opioids, and amphetamines were substantially higher than that for laughing gas (*p* < 0.001), while the difference for cannabinoids was not statistically significant (*p* = 0.057).

### 3.5. Univariate Analysis

As shown in Table 3, participants having any previous knowledge of N_2_O as recreational substance were more frequently of male gender than those having no previous knowledge (47.8% vs. 32.0%, *p* = 0.004) and were also substantially younger (39.85 ± 7.25 years vs. 43.86 ± 9.66 years, *p* < 0.001) and less experienced as medical professionals (13.33 ± 7.68 years vs. 17.24 ± 9.94 years, *p* < 0.001). Similarly, among individuals aged 50 years or more, the proportion of any knowledge of N_2_O abuse was 3.5% compared to 21.0% among those not reporting any understanding (*p* < 0.001). The corresponding proportions were 15.7% vs. 34.5% among professionals having a seniority ≥ 20 years at the time of the survey (*p* < 0.001). When dealing with medical specialties reported by study participants, those having any knowledge of N_2_O abuse more frequently had a background in neurology (25.2% vs. 11.0% not reporting any specific understanding of N_2_O abuse) and emergency medicine (15.7% vs. 4.6%). While the share of general practitioners with or without any previous understanding of N_2_O abuse was similar (18.3% and 17.8%, respectively), any knowledge of N_2_O recreational use was less frequently reported among professionals with a background in psychiatry (11.3% vs. 33.5%) and internal medicine (9.6% vs. 12.8%). No differences were reported by regions where the participant lived and/or worked or by information source (chi-square test, *p* < 0.001).

**Figure A1:**
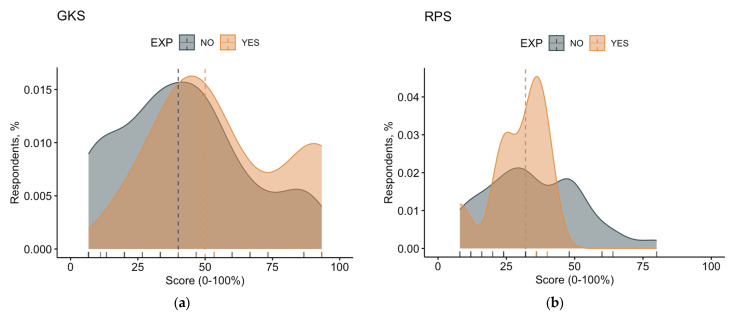
Density plots for general knowledge score (**a**) and risk perception score (**b**) of 115 Italian physicians participating in the survey on knowledge, attitudes, and practices on N_2_O abuse, as broken down by having or not any experience (EXP) on managing a N_2_O abuse case. Not significantly higher GKS estimates were reported among participants having previously managed any case of N_2_O abuse compared to those having not (53.89% ± 25.70 vs. 43.08% ± 24.08; Mann–Whitney U 1341.5, *p* = 0.083). On the contrary, RPS was lower among subjects with any previous experience than among those without (29.17% ± 10.11 vs. 34.86% ± 17.45; Mann–Whitney U 993.0, *p* = 0.268).

Focusing on participants having any understanding of N_2_O abuse, GKS and RPS were not statistically correlated (rho = −0.082, *p* = 0.386), and the highest GKS estimates were reported among participants having previously managed any case of N_2_O abuse (53.89% ± 25.70 vs. 43.08% ± 24.08; Mann–Whitney U 1341.5, *p* = 0.083). On the contrary, the RPS estimate was lower among these subjects than among unexperienced ones (29.17% ± 10.11 vs. 34.86% ± 17.45; Mann–Whitney U 993.0, *p* = 0.268) (Appendix A Figure A1). When RPS and GKS were compared by the medical background of sampled participants (Table 4), the highest knowledge status was reported by general practitioners (53.33% ± 33.67), followed by specialists in emergency medicine (51.48% ± 12.27), neurology (50.11% ± 23.54), internal medicine (38.18% ± 6.73), and other specialties (33.91% ± 19.25) and with the lowest estimates for specialists in psychiatry. By assuming a specialty in internal medicine as the reference category, the difference was significant for all other categories but psychiatry (*p* = 0.745). Focusing on RPS, the highest estimate was associated with the descriptive category of “other specialties” (45.80% ± 25.77), followed by psychiatry (35.08% ± 19.88), neurology (34.33% ± 18.44), general practitioners (34.10% ± 10.93), internal medicine (32.00% ± 14.31), and eventually emergency medicine (31.71% ± 14.09). However, all differences were not statistically significant (*p* > 0.05 for all estimates).

The outcome variable of having or having not previously managed any N_2_O case by the individual characteristics of the participating physicians is reported in Table 5. Briefly, reporting N_2_O abuse as a severe or even very severe condition was the frequent response from individuals without any previous experience in the managing of N_2_O abuse cases compared to experienced individuals (59.3% vs. 29.2%, *p* = 0.016), while no significant differences were reported when dealing with F^N2O^ and the cumulative RPS.

On the contrary, having a previous experience with managing N_2_O abuse was associated with perceiving N_2_O abuse cases as potentially affecting daily working activities (33.3% vs. 6.6%, *p* = 0.001). Finally, participants relying on formation courses for their professional update more frequently reported having not experienced N_2_O cases compared to among experienced ones (92.3% vs. 70.8%, *p* = 0.012).

### 3.6. Multivariable Analysis

**Figure 4:**
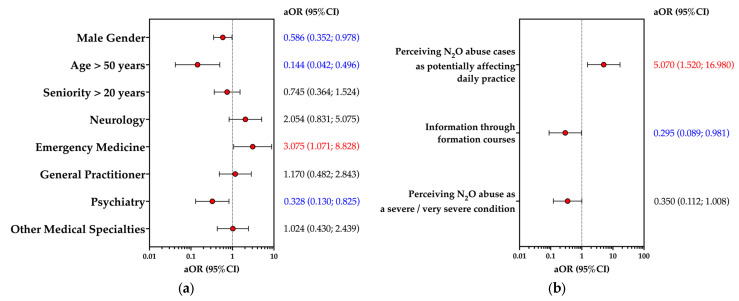
Forest plot for adjusted odds ratios (aOR) and their respective 95% confidence intervals (95%CI) for having any previous knowledge of N_2_O as an abuse substance (all participants, N = 396; subfigure (**a**)) and having previously managed any N_2_O abuse case (only participants having any knowledge of N_2_O as an abuse substance, N = 115, subfigure (**b**)).

The results of multivariable analysis are reported in Figure 4. Focusing on model I (outcome variable: having any knowledge of N_2_O as a recreational substance), all participants of the final sample were included, and the following explanatory variables were taken into account: gender, age > 50 years, and medical specialty. As shown in Figure 4a, having any knowledge of N_2_O as an abuse substance was significantly associated with working in the emergency department (aOR 3.075; 95%CI 1.071 to 8.828). Male gender (aOR 0.586; 95%CI 0.352 to 0.978), age > 50 years (aOR 0.144; 95%CI 0.042 to 0.496), and having an occupational background in psychiatry (aOR 0.328; 95%CI 0.130 to 0.825) were characterized as negative predictors of the outcome variable.

Model II (outcome variable: having previously managed any N_2_O abuse case; only participants with any previous understanding of N_2_O abuse, N = 115) included the following explanatory variables: agreeing or strongly agreeing with the perception of N_2_O abuse as potentially affecting daily practices, relying on formation courses for their medical update, and perceiving N_2_O abuse as a severe/very severe condition. The outcome variable was positively associated with the perceived burden on daily practice of N_2_O abuse (aOR 5.070; 95%CI 1.520 to 16.980) and negatively with relying on formation courses (aOR 0.295; 95%CI 0.089 to 0.981).

## 4. Discussion

### 4.1. Summary of Key Results

Through the present cross-sectional study, we collected data about the knowledge, attitudes, and practices on N_2_O abuse from a convenience sample of 396 Italian medical professionals. In fact, only 29.04% of them had any knowledge that N_2_O has recreational uses: belonging to younger age groups and reportedly working in the emergency department were predictive of a better awareness about N_2_O abuse, while having an occupational background in psychiatry was less frequently associated with a proper understanding of this potential issue. An even smaller proportion (a total of 24 participants, that is, 6.06% of the total sample) had any personal experience in the managing of N_2_O abuse cases.

Despite repeated claims about the increased recreational use of N_2_O in the EU-EEA (particularly in Northern Europe and France) [8,11,30,35,36] and in mainland China [34], until recently, N_2_O abuse has a remained a substantially forgotten topic. On the one hand, N_2_O is legally available in most European countries as well as in the USA and mainland China because of the wide range of its legal uses [1,8,11,30]. While national and international directives for the vending, storage, transport, and labelling of hazardous chemicals have eventually regulated and limited the intended uses of N_2_O, all unintended recreational uses are not covered, leaving N_2_O trade for recreational use quite difficult to track, counter, and prosecute [30]. As a consequence, estimating the actual prevalence of N_2_O abuse is difficult, as it is often limited to some cross-sectional studies [37,56]. To the best of our knowledge, no specific estimate is available to date for Italy [8]. In this regard, it should be stressed that our study was not specifically designed for providing any hints as to the Italian prevalence of N_2_O abuse. As a consequence, translating the reported previous interaction with N_2_O abuse cases from 6.06% of participants into a proxy for the prevalence estimate would be not only inappropriate but also somehow incautious. For instance, because of the design of this study, we are unable to rule out that several participants may have personal experience with the very same index patient or that this expertise was obtained in foreign countries. However, our results collectively suggest that the recreational use of N_2_O does occur and that Italian health authorities do not properly track incident cases.

While the lack of Italian data is therefore reasonably due to the lack of an appropriate reporting system, our data suggest that such an information gap may be reasonably associated with the improper awareness of medical professionals involved in the management of incident cases. Compared to opioids and cocaine, N_2_O does not elicit substantial dependence, but clinical consequences of its chronic abuse are significant [2,3,4,5,13,28,29]. Through inhibition of vitamin B12, N_2_O abuse eventually results in a wide range of signs and symptoms, including hematological disorders such as anemia and thrombophilia (with increased risk for deep vein thrombosis and pulmonary embolism), sensorimotor peripheral neuropathies, and mood disorders. As all aforementioned conditions are far from uncommon causes of medical consultations and hospital admissions [1,3,12], until specific biomarkers of N_2_O abuse are made extensively available [12], the accurate identification of incident cases will be based on the clinical suspicion when facing patients characterized by vague but quite consistent neurological complications and, more specifically, the bilateral and symmetrical limb numbness that mostly evolves to glove and sock paresthesia [1,2,3,4,5,12,13,20,28,29,56,57,58,59,60,61,62]. In this regard, our study suggests that frontline professionals including general practitioners, specialists from emergency departments, and also consultants from the fields of neurology and psychiatry could fail to properly diagnose incident cases due to a mixture of inappropriate knowledge status and low risk perception. Indeed, the knowledge gaps we identified did encompass nearly all aspects of N_2_O abuse from epidemiological data to the main clinical features. Even though some medical professionals performed quite better than other specialists, with better GKS estimates from general practitioners, specialists in emergency medicine, and neurologists [1,12,30,63], the overall estimates remained quite unsatisfying. This is particularly upsetting when dealing with results from specialists in psychiatry: As cases of N_2_O abuse may be characterized by mood disorders and even severe psychosis [1,3,28,58,59], psychiatrists could be involved in the first stages of the medical assessment of the chronic abuse of laughing gas, and their lack of familiarity with N_2_O abuse could impair the proper characterization of incident cases.

The analysis of factors associated with having or having not managed a case of N_2_O abuse led to even more baffling results.

On the one hand, having managed a case of N_2_O abuse was significantly associated with the perception of the potential impact on daily practice (aOR 5.070; 95%CI 1.520 to 16.980). These estimates are consistent with the potential severity and complicated management of incident cases due to a series of factors that could be underscored by professionals unfamiliar with N_2_O abuse [1,12,20,64,65,66]. For one, even though the large majority of N_2_O users does not reasonably develop any significant complication, a reduced but noticeable proportion of cases can be complicated by hypercoagulability associated with secondary hyperhomocysteinemia [1,20,64,65,66,67,68,69], leading to deep vein thrombosis and potentially life-threatening conditions such as cerebral venous sinus thrombosis [13,66,70,71,72] and pulmonary embolism [64,67,68,69]. Second, the available data suggest that a substantial share of incident cases eventually fails to cope with rehabilitation for a variety of different causes, including the lack of support from familiars of social services or a pre-existing, complicated background of multiple substance abuse [14,23,73,74,75,76]. Third, even patients who receive an appropriate and timely diagnosis and benefit from an appropriate medical follow-up often fail or at least struggle to fully recover from N_2_O-induced sensorimotor neuropathy and the resulting ataxia [24,26,29,73,74,76,77,78,79,80,81].

On the other hand, relying on formation courses for medical update was negatively associated with having previously managed cases of N_2_O (aOR 0.350; 95%CI 0.098 to 0.981), and again, some tentative explanations can be provided. According to the design of the present study, only participants having any pre-existing knowledge of N_2_O abuse were asked about their previous personal expertise. As a consequence, the subgroup of participants who received the full questionnaire included a group of professionals who had developed their awareness about N_2_O mostly due to their daily practice and a far larger group of participants who never met any case of N_2_O abuse and only relying on formation courses for their awareness of this potential health threat, whose extensive and adequate analysis was still quite limited, as suggested by the proportion of participants claiming any previous information about N_2_O abuse. In other words, the role of formation courses should be reasonably evaluated in a fairly positive way as instrumental in improving the overall awareness of medical professionals [52,82,83,84], as previously stressed by similarly designed cross-sectional studies [42,46,85].

### 4.2. Limitations

Our study is affected by several limitations that collectively affect the generalizability of the collected data. First, in order to reduce turnaround time and quickly reach the targeted sample size, the study was designed as a web-based survey. Despite the acknowledged reliability and cost effectiveness, this design is affected by several shortcomings. Among the main limitations of web-based studies, the most notable one is represented by a certain “self-selection” of participants [43,85,86,87,88,89,90], with the eventual oversampling of certain subgroups, particularly those subjects with a greater familiarity with the internet and social media and the resulting attitude toward sharing personal information [42,43,44,87]. For instance, despite the recent impact of the “great resignation phenomenon” [83,91], the Italian medical workforce includes a large share of individuals aged 50 years or more [92,93], which in our study did not exceed a very limited proportion of 15.91%, limiting the eventual representativity of the final sample over the total population of Italian medical professionals. Moreover, it is reasonable that the very same participation in an internet discussion group and the resulting attitude toward sharing individual data may have led to the further, preventive selection of targeted professionals, supporting a very cautious interpretation of our results, even in general terms. Nonetheless, the participating subjects were reasonably more familiar with the assessed topic than those not participating in the study, with the eventual oversampling of individuals with higher understanding of that specific theme [42,43,94,95]. This potential bias is otherwise suggested by the proportion of participants reporting any previous awareness of N_2_O abuse. Even though it encompassed less than 29.02% of participating individuals, this would mean that around 1/3 of Italian physicians has any knowledge of a condition that still remains substantially not reported [8]. In other words, the present study was hardy generalizable, particularly in a country such as Italy, which is characterized by distinctive regional patterns, and considering school-specific training during the residency programs [88], as otherwise stressed by the reduced number of participants claiming to have received a previous university-level formation on N_2_O abuse.

**Figure A2:**
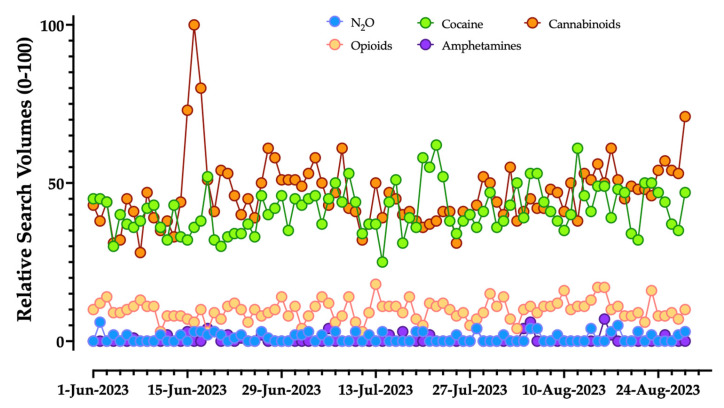
Relative search volumes reported in normalized percent volumes, as provided by Google Trends^TM^ for the search terms of “laughing gas” (Italian: “gas esilarante”), “cocaine” (Italian: “cocaina”), “cannabinoids” (Italian: “cannabis”), “opioids” (Italian: “eroina” and “oppioidi”), and “amphetamines” (Italian: “amfetamine”).

Second, a cross-sectional study is not designed for properly assessing the causal relationships between the assessed risk factors or explanatory conditions and the targeted outcome [37,56,96,97]. A cross-sectional design may be considered as well suited for studying the acceptance of interventions where the outcome variable (e.g., vaccination status) is only partially limited by time [98,99], while potential explanatory variables pre-exist the delivery of that intervention [100,101,102]. In this case, not only are we unable to actually discriminate between participants having obtained their actual understanding of N_2_O poisoning by formal education, medical education, or personal experience, but we should also stress that a significant role was possibly played by a factor quite difficult to ascertain, such as media coverage at the time of the survey [42,98,103,104]. Uncontrolled media claims about a misunderstood topic such as a N_2_O could possibly contribute to the knowledge gap and misunderstanding associated with the false belief of being “informed” about that topic, and a similar phenomenon has been described in previous KAP studies about the medical workforce [42,46,99,105]. As a proxy of the media coverage on N_2_O abuse, we specifically analyzed the relative search volumes on this search term as provided by Google Trends™ [106,107,108,109]. Google Trends^TM^ is the open-access online tool provided by Alphabet that reports the overall queries on a certain keyword as the normalized ratio over the total of web queries in that specific timeframe [110,111,112]. As shown in Appendix A Figure A2, during the study period, the overall queries about N_2_O remained quite marginal when compared to other abuse substances such as cocaine, opioids, amphetamines, and cannabinoids.

Third, the fulfillment of inclusion criteria, most notably the qualification as a medical professional working in a certain specialty or sub-specialty, was not specifically validated for each participant during and/or after the collection of the questionnaire. As a consequence, we cannot rule out that some of the respondents did not fully adhere to our selection criteria. However, the questionnaire was delivered in a closed medical community, and only individuals having been preventively confirmed as registered medical professionals by the website and discussion group managers were admitted [40]. In other words, despite its potential significance, this issue was somehow limited or at least did not furtherly impair the representativity of our study compared to the whole of the Italian medical workforce.

**Figure A3:**
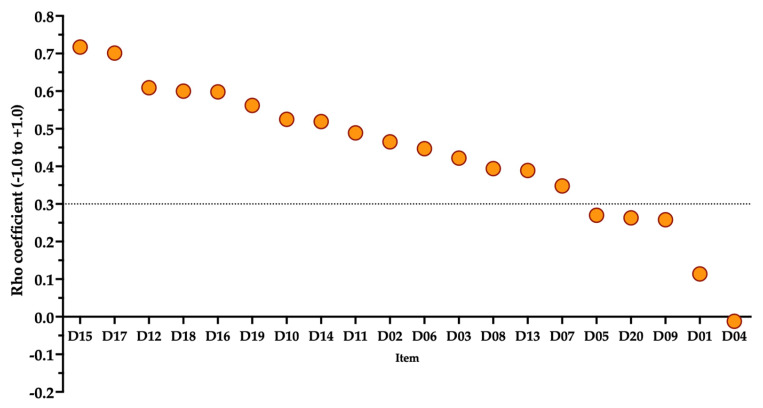
Graphic representation of the items included in the knowledge test by their correlation (reported as rho coefficient) with the cumulative number of corrected answers. According to the model proposed by Möltner and Jünger [51,52], only items having a rho > 0.3 (i.e., all items above the dotted line) were eventually included in the general knowledge score (GKS).

Fourth, the general knowledge test was specifically designed for this study. Even though the items included in the questionnaire were derived from the retrospective analysis of the available evidence on nitrous oxide abuse [1,2,3,4,5,12,13,20,24,28,29,76,113], most notably the recent report from ECMDDA [8], it could require further revisions and adjustments: As a consequence, the present report must be acknowledged as a preliminary one. Not coincidentally, the original questionnaire (only delivered to the 15 beta testers not included in the present survey) included a total of 27 items. Moreover, 7 of them were removed during the beta testing, and when the approach suggested by Möltner and Jünger was applied [51,52], 5 out of the 20 items included in the final version of the questionnaire were removed from the calculation of GKS as not truly representative of the actual understanding of the participant about the inquired topic (Appendix A Figure A3). On the other hand, our actual understanding of the clinical and laboratory features of N_2_O poisoning is not only improving over time following the increasing number of cases reported in the international medical literature [1,2,3,4,5,12,13,20,28] but is reasonably biased by the nature of the available reports. The large majority of published studies on N_2_O abuse are represented by case reports, and we cannot rule out that the available base of evidence may be affected by the over-representation of uncommon and severe features over more vague and unclear signs and symptoms [1,12,20]. Future iterations of our study could therefore benefit not only from the increasing base of evidence on N_2_O poisoning among recreational abusers but also from a more restrictive choice of items included in the knowledge test, e.g., by means of the calculation of the content validity ratio from subject matter experts [114].

Our study should, therefore, be regarded as a pilot one, whose most significant contributions to public health professionals are represented by (1) having made available a preliminary quantification of the KAP of Italian physicians about N_2_O abuse and (2) having suggested that Italian cases of N_2_O poisoning do occur and are not reasonably recorded because of the lack of an appropriate reporting system.

## 5. Conclusions

In conclusion, our study suggests that the Italian physicians potentially involved in the early management of N_2_O cases could benefit from specifically tailored interventions aimed to raise their capability to cope with a rapid and appropriate diagnosis of this abuse of growing interest at the European level. Despite the limits of the present study, our methodology could be implemented in future research monitoring the knowledge status of medical professionals towards N_2_O and similarly emerging substances of abuse.

## Figures and Tables

**Table 1:** Characteristics of the 396 Italian physicians participating in the survey on the knowledge, attitudes, and practices on nitrous oxide (N_2_O) abuse (Note: SD = standard deviation).

Variable	Total (N/396, %)	Average ± S.D.
Gender		
Male	145, 36.62%	
Female	251, 63.38%	
Age (years)		42.70 ± 9.71
Age ≥ 50 years	63, 15.91%	
Seniority (years)		16.10 ± 9.47
Seniority ≥ 20 years	115, 29.04%	
Medical specialty		
Internal medicine	47, 11.87%	
Neurology	60, 15.15%	
Emergency medicine	31, 7.83%	
General practitioner	71, 17.93%	
Psychiatry	107, 27.02%	
Other	80, 20.20%	
Living in …		
Northern Italy ^1^	197, 49.75%	
Central Italy ^2^	115, 29.04%	
Southern Italy ^3^	54, 13.64%	
Major islands ^4^	30, 7.58%	
Reporting any knowledge of N_2_O abuse	116, 29.04%	
Information source		
Formation courses	332, 83.84%	
Official websites	326, 82.32%	
Non-official websites	55, 13.89%	
Medical journals	143, 36.11%	
Other healthcare workers	225, 56.82%	
Social media	43, 10.86%	
Friends and/or relatives	0, -	

^1^ Aosta Valley, Piedmont, Liguria, Lombardy, Veneto, Autonomous Province of Trento, Autonomous Province of Bolzano, Friuli-Venezia-Giulia, and Emilia Romagna; ^2^ Tuscany, Umbria, Marche, and Lazio; ^3^ Campania, Abruzzo, Apulia, Basilicata, and Calabria; ^4^ Sicily and Sardinia.

**Table 2:** Attitudes and practices reported by 115 Italian physicians having any previous knowledge about nitrous oxide (N_2_O) abuse.

Variable	Total (N/115, %)
Knowledge status	
General knowledge score (average ± SD)	45.33% ± 24.71
Median value	46.67%
General knowledge score > median (46.67%)	40, 34.78%
Risk perception	
Acknowledging N_2_O abuse as a frequent/very frequent condition	10, 8.70%
Acknowledging N_2_O abuse as a severe/very severe condition	61, 53.04%
Risk perception score (average ± SD)	33.67% ± 16.32
Median value	32.00%
Risk perception score > median (32.00%)	51, 44.35%
Perceiving N_2_O abuse cases as a likely occurrence during daily activity (agree/totally agree)	19, 16.52%
Perceiving N_2_O abuse cases as potentially affecting daily working activities (agree/totally agree)	14, 12.17%
Confident of being able to recognize a N_2_O abuse cases (agree/totally agree)	11, 9.56%
Having previously managed any N_2_O abuse case	24, 20.87%
Any information on N_2_O abuse	27, 23.48%

**Table 3:** Characteristics of the 396 Italian physicians participating in the survey on knowledge, attitudes, and practices on nitrous oxide (N_2_O) abuse by their previous knowledge of N_2_O abuse (Note: SD = standard deviation). Analyses were performed by means of chi-square test with continuity correction for categorical values and by means of Mann–Whitney test for continuous variables.

Variable	Participants by Their Knowledge of N_2_O Abuse		*p*-Value
	Any Knowledge (N/115)	No Knowledge (N/281)	
Gender			
Male	55, 47.8%	90, 32.0%	0.004
Female	60, 52.2%	191, 68.0%	
Age (years)	39.85 ± 7.25	43.86 ± 9.66	0.001
Age ≥ 50 years	4, 3.5%	59, 21.0%	<0.001
Seniority (years)	13.33 ± 7.68	17.24 ± 9.94	0.001
Seniority ≥ 20 years	18, 15.7%	96, 34.5%	<0.001
Medical specialty			<0.001
Internal medicine	11, 9.6%	36, 12.8%	
Neurology	29, 25.2%	31, 11.0%	
Emergency medicine	18, 15.7%	13, 4.6%	
General practitioner	21, 18.3%	50, 17.8%	
Psychiatry	13, 11.3%	94, 33.5%	
Other	23, 20.0%	57, 20.3%	
Living in …			0.065
Northern Italy ^1^	60, 52.2%	137, 48.8%	
Central Italy ^2^	40, 34.8%	75, 26.7%	
Southern Italy ^3^	9, 7.8%	45, 16.0%	
Major islands ^4^	6, 5.2%	24, 8.5%	
Information source			
Formation courses	101, 87.8%	231, 82.2%	0.219
Official websites	90, 78.3%	236, 84.0%	0.226
Non-official websites	13, 11.3%	42, 14.9%	0.429
Medical journals	41, 35.7%	102, 36.3%	0.995
Other healthcare workers	63, 54.8%	162, 57.7%	0.681
Social media	16, 13.9%	27, 9.6%	0.284

Note: ^1^ = including the following Italian regions: Valle d’Aosta, Piedmont, Liguria, Emilia-Romagna, Lombardy, Autonomous Provinces of Trento and Bolzano, Veneto, Friuli Venezia Giulia; ^2^ = including the following Italian regions: Tuscany, Umbria, Marche, Latium; ^3^ = including the following Italian regions: Abruzzo, Campania, Molise, Apulia, Basilicata, Calabria; ^4^ = including the following Italian regions: Sicily, Sardinia.

**Table 4:** Comparison of general knowledge score (GKS) and risk perception score (RPS) from a sample of Italian physicians (N = 115) having any understanding of N_2_O. Comparisons were performed by means of Kruskal–Wallis (K–W) tests and by assuming participants with a medical background in internal medicine as the reference group.

	GKS (Average ± SD)	*p*-Value	RPS (Average ± SD)	*p*-Value
Internal medicine	38.18% ± 6.73	REFERENCE	32.00% ± 14.31	REFERENCE
Neurology	50.11% ± 23.54	0.003	34.33% ± 18.44	0.075
Emergency medicine	51.48% ± 12.27	0.001	31.71% ± 14.09	0.085
General practitioner	53.33% ± 33.67	0.003	34.10% ± 10.93	0.058
Psychiatry	18.46% ± 9.87	0.006	35.08% ± 19.88	0.080
Other specialties	33.91% ± 19.25	0.745	45.80% ± 25.77	0.056

**Table 5:** Characteristics of Italian physicians (N = 115) having any understanding of N_2_O by their personal experience in the managing of N_2_O abuse cases (any vs. never).

Variable	Previous Personal Experience in the Managing of N_2_O Abuse Cases		*p*-Value
	Any (N/24, %)	Never (N/91, %)	
Gender			0.353
Male	14, 58.3%	41, 45.1%	
Female	10, 41.7%	50, 54.9%	
Age ≥ 50 years	0, -	4, 4.4%	0.675
Seniority ≥ 20 years	4, 16.7%	14, 15.4%	1.000
Living in…			0.068
Northern Italy ^1^	17, 70.8%	43, 47.3%	
All other regions ^2^	7, 29.2%	48, 52.7%	
Medical specialty			0.634
Internal medicine	3, 12.5%	8, 8.8%	
Neurology	3, 12.5%	26, 28.6%	
Emergency medicine	4, 16.7%	14, 15.4%	
General practitioner	4, 16.7%	17, 18.7%	
Psychiatry	4, 16.7%	9, 9.9%	
Other	6, 25.0%	17, 18.7%	
Higher knowledge status	10, 41.7%	30, 33.0%	0.579
Higher risk perception score	11, 45.8%	40, 44.0%	1.000
Acknowledging N_2_O abuse as a frequent/very frequent condition	0, -	10, 11.0%	0.196
Acknowledging N_2_O abuse as a severe/very severe condition	7, 29.2%	54, 59.3%	0.016
Any information on N_2_O abuse	7, 29.2%	20, 22.0%	0.639
Perceiving N_2_O abuse cases as a likely occurrence during daily activity (agree/totally agree)	4, 16.7%	15, 16.5%	1.000
Perceiving N_2_O abuse cases as potentially affecting daily working activities (agree/totally agree)	8, 33.3%	6, 6.6%	0.001
Confident of being able to recognize N_2_O abuse cases (agree/totally agree)	4, 16.7%	7, 7.7%	0.347
Information source			
Formation courses	17, 70.8%	84, 92.3%	0.012
Official websites	20, 83.3%	70, 76.9%	0.690
Non-official websites	0, -	13, 14.3%	0.109
Medical journals	11, 45.8%	30, 33.0%	0.352
Other healthcare workers	10, 41.7%	53, 58.2%	0.222
Social media	3, 12.5%	13, 14.3%	1.000

^1^ Aosta Valley, Piedmont, Liguria, Lombardy, Veneto, Autonomous Province of Trento, Autonomous Province of Bolzano, Friuli-Venezia-Giulia, and Emilia Romagna; ^2^ Tuscany, Umbria, Marche, Lazio, Campania, Abruzzo, Apulia, Basilicata, Calabria, Sicily, and Sardinia.

## Data Availability

Data are available on request. Anonymized and collective raw data are available on request to the corresponding author.

## Supplementary Materials

The following supporting information can be downloaded at: https://www.mdpi.com/article/10.3390/medicina59101820/s1, Supplementary File S1: Translation of Gazzetta Ufficiale no. 76, dated 31 March 2008.

## Appendix A

**Table A1:** STROBE Statement—Checklist of items that should be included in reports of cross-sectional studies.

	Item No.	Recommendation	Page
**Title and abstract**	1	(a) Indicate the study’s design with a commonly used term in the title or the abstract.	1
		(b) Provide in the abstract an informative and balanced summary of what was done and what was found.	1
**Introduction**			
Background/rationale	2	Explain the scientific background and rationale for the investigation being reported.	1–3
Objectives	3	State specific objectives, including any prespecified hypotheses.	2–3
**Methods**			
Study design	4	Present key elements of study design early in the paper.	3
Setting	5	Describe the setting, locations, and relevant dates, including periods of recruitment, exposure, follow-up, and data collection.	3
Participants	6	(a) Give the eligibility criteria, and the sources and methods of selection of participants.	3–4
Variables	7	Clearly define all outcomes, exposures, predictors, potential confounders, and effect modifiers. Give diagnostic criteria, if applicable.	3–4
Data sources/measurement	8	For each variable of interest, give sources of data and details of methods of assessment (measurement). Describe comparability of assessment methods if there is more than one group.	4–6
Bias	9	Describe any efforts to address potential sources of bias.	4–5
Study size	10	Explain how the study size was arrived at.	4
Quantitative variables	11	Explain how quantitative variables were handled in the analyses. If applicable, describe which groupings were chosen and why.	5–6
Statistical methods	12	(a) Describe all statistical methods, including those used to control for confounding.	5–6
		(b) Describe any methods used to examine subgroups and interactions.	5–6
		(c) Explain how missing data were addressed.	5–6
		(d) If applicable, describe analytical methods, taking account of sampling strategy.	5–6
		(e) Describe any sensitivity analyses.	-
**Results**			
Participants	13	(a) Report numbers of individuals at each stage of study, e.g., numbers potentially eligible, examined for eligibility, confirmed eligible, included in the study, completing follow-up, and analyzed.	6
		(b) Give reasons for non-participation at each stage.	6
		(c) Consider use of a flow diagram.	6
Descriptive data	14	(a) Give characteristics of study participants (e.g., demographic, clinical, and social) and information on exposures and potential confounders.	6–7
		(b) Indicate number of participants with missing data for each variable of interest	6–7
Outcome data	15	Report numbers of outcome events or summary measures.	8–10
Main results	16	(a) Give unadjusted estimates and, if applicable, confounder-adjusted estimates and their precision (e.g., 95% confidence interval). Make clear which confounders were adjusted for and why they were included.	10–13
		(b) Report category boundaries when continuous variables were categorized.	10–13
		(c) If relevant, consider translating estimates of relative risk into absolute risk for a meaningful time period.	-
Other analyses	17	Report other analyses performed, e.g., analyses of subgroups and interactions and sensitivity analyses.	10–13, Appendix A Table A7, Appendix A Figure A1, Figure A2 and Figure A3
**Discussion**			
Key results	18	Summarize key results with reference to study objectives.	13–15
Limitations	19	Discuss limitations of the study, taking into account sources of potential bias or imprecision. Discuss both direction and magnitude of any potential bias.	15–16
Interpretation	20	Give a cautious overall interpretation of results considering objectives, limitations, multiplicity of analyses, results from similar studies, and other relevant evidence.	13–16
Generalizability	21	Discuss the generalizability (external validity) of the study results.	13–14
**Other information**			
Funding	22	Give the source of funding and the role of the funders for the present study and, if applicable, for the original study on which the present article is based.	17

**Table A2:** Authors’ translation of the informed consent.

Informed Consent. Estimated colleague, the present survey has been developed and shared with the aim to assess the knowledge, attitudes, and practices of medical professionals on the N_2_O abuse in Italy. Alongside vaccination practice, preventive practices against tick bites will also be inquired about. The present survey has only scientific aims. No economic or similar compensation is guaranteed to the participants.
While we thank you for your cooperation, we stress that web-based surveys must fulfill the requirements represented by the “Helsinki protocol” and EU Regulation 2016/679. In order to fulfill the requirements of the Helsinki protocol, we are formally requesting your consent. Without your consent, the survey will not continue. Even after your consent, you can leave the present survey at any moment, until the sharing of the questionnaire (button “share module” at the end of the questionnaire). Moreover, we stress that the questionnaire will be registered in anonymous form, and in no way could it be associated with the compiler, as we will not retain any specific, individual information (e.g., signature, personal address, etc.). All requested personal data are generic ones and functional to the demographic analyses (gender, age, etc.).
According to the EU Regulation 2016/279 (GDPR), we also state the following: (1) The data controller and processor as well as the party responsible for their retention during the analyses will be Dr. **********, whom you can ask about the process through his personal email (*********). Collected data are generic ones, with solely scientific aims that have been previously reported. Please be aware that all personal data must be shared with Criminal Law Authorities; without a previous personal consent, in the cases that are specifically reported by the current legal framework and without a specific request, the retrieved data will not be shared with third parties. (2) After the completion of the questionnaire, we cannot identify in any way the compiler; as the questionnaire is totally anonymous by design, we cannot perform any modification, correction, or removal of the data collected. Data will be retained only for the time strictly required for the aforementioned analyses.

### Appendix A.1. Authors’ Translation of the Questionnaire

(**Note:** The questionnaire was originally designed and delivered in Italian; the present English translation of the questionnaire was carried out by study authors in order to provide a more appropriate ascertainment of the full content of the present study. The accuracy of the present translation was in turn guaranteed by the reverse translation of the items:

Italian items were initially translated in English by study authors (M.R. and M.B.). A colleague that was totally blind regarding the Italian questionnaire was requested to translate the English version into Italian.

The items were then compared with the original version in order to ascertain whether the original content was retained.

**Table A3:** Personal experiences with laughing gas (N_2_O).

	Yes	No	Do Not Remember/I Prefer Not to Answer
Q01. Do you know that N_2_O can be used as a recreational drug?	[ ] (go to Q02)	[ ] (go to Appendix A.2)	[ ] (go to Appendix A.2)
Q02. During your medical practice, did you manage any case of N_2_O abuse?	[ ]	[ ]	[ ]
Q03. During your studies, did you receive any University-level formation on N_2_O?	[ ]	[ ]	[ ]

**Table A4:** Level of your knowledge (please mark the right statement).

	Your Answer		
D1. Since 2010, several countries have experienced an increasing prevalence of N_2_O abuse.	True	False	Do not know
D2. N_2_O abuse is usually identified through urinary biomarkers.	True	False	Do not know
D3. The number of reported N_2_O poisonings in countries such as the Netherlands and the United Kingdom has increased more than 10 times in the last 10 years.	True	False	Do not know
D4. Abuse of N_2_O through use of cartridges and canisters can cause lung barotrauma.	True	False	Do not know
D5. Inhalation of N_2_O can be associated with frost bite of the respiratory airways, mouth, and lips.	True	False	Do not know
D6. Upon inhalation, N_2_O has a latency of around 10 min.	True	False	Do not know
D7. Chronic side effects are usually severe and long-lasting.	True	False	Do not know
D8. Abuse of N_2_O does impair the body balance and driving ability.	True	False	Do not know
D9. Lung barotrauma is the main cause of death following abuse of N_2_O.	True	False	Do not know
D10. N_2_O irreversibly inactivates vitamin B12.	True	False	Do not know
D11. N_2_O causes sedation.	True	False	Do not know
D12. In cases of chronic toxicity from N_2_O abuse, most patients complain of sensorimotor symptoms.	True	False	Do not know
D13. Chronic N_2_O abuse is associated with an increased risk of deep vein thrombosis and pulmonary embolism.	True	False	Do not know
D14. Complications associated with chronic N_2_O abuse are at least partially reversible through a prompt diagnosis.	True	False	Do not know
D15. Recreational abuse of N_2_O is more frequently reported among individuals aged > 25 years than among 15- to 24 year-old subjects.	True	False	Do not know
D16. In the last decades, the recreational abuse of N_2_O among high school students from Northern European countries and France had a lifetime prevalence ranging between…	1% to 5% 6% to 15% 16% to 25% 26% to 35% Do not know		
D17. Chronic abuse of N_2_O is frequently associated with magnetic resonance imaging anomalies of the…	Cerebral cortex Brainstem Spinal cord, cervical tract Spinal cord, thoracic tract Do not know		
D18. Symptoms associated with chronic abuse of N_2_O can be enhanced by…	Surgery of the esophagus Surgery of the stomach and biliary system Surgery of liver and pancreas Surgery of small bowel and large bowel		
D19. Symptoms associated with chronic abuse of N_2_O can be enhanced by pregnancy.	True	False	Do Not know
D20. Paresthesia associated with chronic N_2_O abuse is usually described as…	Distal (“sock”) Proximal (“glove”) Glove and sock, bilateral and symmetrical Glove and sock, unilateral and/or asymmetrical		

**Table A5:** Perceptions and beliefs about laughing gas (N_2_O).

**Q04. According to your current understanding, how do you perceive N_2_O abuse in Italy:**	
Regarding its SEVERITY	(1) Not significant at all (2) (3) (4) (5) very significant
Regarding its FREQUENCY	(1) Not significant at all (2) (3) (4) (5) very significant
**Q05. How would you rate the health threat represented by**	
N_2_O abuse	Not significant at all (1) (2) (3) (4) (5) (6) (7) (8) (9) (10) Very Significant
Cocaine abuse	Not significant at all (1) (2) (3) (4) (5) (6) (7) (8) (9) (10) Very Significant
Opioid abuse	Not significant at all (1) (2) (3) (4) (5) (6) (7) (8) (9) (10) Very Significant
Cannabinoid abuse	Not significant at all (1) (2) (3) (4) (5) (6) (7) (8) (9) (10) Very Significant
Amphetamine abuse	Not significant at all (1) (2) (3) (4) (5) (6) (7) (8) (9) (10) Very Significant
**Q06. From your point of view, in the following 12 months, N_2_O abuse**	
(1) …will be a likely occurrence during daily activities.	
Totally disagree	[ ]
Disagree	[ ]
Neutral	[ ]
Agree	[ ]
Totally agree	[ ]
(2) …will significantly affect your daily activities.	
Totally disagree	[ ]
Disagree	[ ]
Neutral	[ ]
Agree	[ ]
Totally agree	[ ]
**Q07. Are you confident of being able to recognize incident N_2_O abuse cases during your daily activities?**	
Totally disagree	[ ]
Disagree	[ ]
Neutral	[ ]
Agree	[ ]
Totally agree	[ ]

### Appendix A.2. Some Personal Information about You

**A01**. Your age: _________ (years)
**A02**. Your seniority as a physician: __________ (years)
**A03**. Gender: M [ ] F [ ]; Prefer not to answer [ ]
**A04**. Region of residence:

[ ] Northern Italy	Aosta Valley, Piedmont, Liguria, Lombardy, Veneto, Autonomous Province of Trento, Autonomous Province of Bolzano, Friuli-Venezia-Giulia, Emilia Romagna
[ ] Central Italy	Tuscany, Umbria, Marche, Lazio
[ ] Southern Italy	Campania, Abruzzo, Apulia, Basilicata, Calabria
[ ] Major islands	Sicily, Sardinia

**A05**. At the moment, you are employed as:
[ ] General practitioner
[ ] Other medical specialty (internal medicine)
[ ] Other medical specialty (neurology)
[ ] Other medical specialty (emergency medicine)
[ ] Other medical specialty (psychiatry)
[ ] Other medical specialty

**A06**. Your main information source(s) on medical topics:
[ ] Formation courses
[ ] Official websites (governmental ones, health authorities, etc.)
[ ] Non-official websites (blogs, personal websites, etc.)
[ ] Medical journals
[ ] Friends, relatives
[ ] Other healthcare workers
[ ] Social media (Facebook, Twitter, etc.)

**Table A6:** **[not for delivery].** Items removed from the final questionnaire.

The actual price per liter of nitrous oxide usually ranges between…	EUR 1.15 to 1.15 EUR 0.50 to 1.15 **EUR 0.05 to 0.15** EUR 0.05 to 0.01 Do not know		
Heavy users of nitrous oxide report the consumption of around 50 or more balloons in a single session or use from a cylinder.	**True**	False	Do not know
Abuse of nitrous oxide has been associated with mass gathering events such as concerts and rave parties.	**True**	False	Do not know
Nitrous Oxide causes endorphins to be released in certain brain regions.	**True**	False	Do not know
Nitrous oxide is a weak greenhouse gas around 300 times less potent than carbon dioxide.	True	**False**	Do not know
Inhalation of five balloons of nitrous oxide could be acknowledged as equivalent to approximately 7 min of nitrous oxide anesthesia.	**True**	False	Do not know
The clandestine or home production of nitrous oxide is less expensive than that commercially available.	True	**False**	Do not know

### Appendix A.3. Supplementary Analyses

**Table A7:** Results of knowledge test from 115 medical professionals participating in the survey on N_2_O abuse (Italy, 2023) ((+) = item correlated with cumulative number of correct answer with rho ≥ 0.300 and eventually included in the calculation of general knowledge score, GKS).

Statement	Answer	No./165, %	Correlation with Higher GKS (rho)
D1. Since 2010, several countries have experienced an increasing prevalence of N_2_O abuse.	TRUE	92, 80.0%	0.114
D2. N_2_O abuse is usually identified through urinary biomarkers.	FALSE	33, 28.7%	0.465 (+)
D3. The number of reported N_2_O poisonings in countries such as the Netherlands and the United Kingdom has increased more than 10 times in the last 10 years.	TRUE	77, 67.0%	0.422 (+)
D4. Abuse of N_2_O through use of cartridges and canisters can cause lung barotrauma.	TRUE	71, 61.7%	−0.012
D5. Inhalation of N_2_O can be associated with frost bite of the respiratory airways, mouth, and lips.	TRUE	77, 67.0%	0.270
D6. Upon inhalation, N_2_O has a latency of around 10 min.	FALSE	39, 33.9%	0.447 (+)
D7. Chronic side effects are usually severe and long-lasting.	FALSE	33, 28.7%	0.348 (+)
D8. Abuse of N_2_O does impair the body balance and driving ability.	FALSE	93, 80.9%	0.394 (+)
D9. Lung barotrauma is the main cause of death following abuse of N_2_O.	FALSE	24, 20.9%	0.258
D10. N_2_O irreversibly inactivates vitamin B12.	TRUE	35, 30.4%	0.525 (+)
D11. N_2_O causes sedation.	FALSE	39, 33.9%	0.489 (+)
D12. In cases of chronic toxicity from N_2_O abuse, most patients complain of sensorimotor symptoms.	TRUE	95, 82.6%	0.609 (+)
D13. Chronic N_2_O abuse is associated with an increased risk of deep vein thrombosis and pulmonary embolism.	TRUE	63, 54.8%	0.389 (+)
D14. Complications associated with chronic N_2_O abuse are at least partially reversible through a prompt diagnosis.	TRUE	69, 60.0%	0.519 (+)
D15. Recreational abuse of N_2_O is more frequently reported among individuals aged > 25 years than among 15- to 24 year-old subjects.	FALSE	50, 43.5%	0.717 (+)
D16. In the last decades, the recreational abuse of N_2_O among high school students from Northern European countries and France had a lifetime prevalence ranging between…	6% and 15%	44, 38.3%	0.598 (+)
D17. Chronic abuse of N_2_O is frequently associated with magnetic resonance imaging anomalies of the…	Spinal cord, cervical tract	26, 22.6%	0.701 (+)
D18. Symptoms associated with chronic abuse of N_2_O can be enhanced by…	Surgery of the stomach and biliary system	17, 14.8%	0.600 (+)
D19. Symptoms associated with chronic abuse of N_2_O can be enhanced by pregnancy.	TRUE	69, 60.0%	0.562 (+)
D20. Paresthesia associated with chronic N_2_O abuse is usually described as…	Glove and sock, bilateral and symmetrical	40, 34.8%	0.263

## References

[1] Marsden P., Sharma A.A., Rotella J.A. (2022). Review Article: Clinical Manifestations and Outcomes of Chronic Nitrous Oxide Misuse: A Systematic Review. EMA Emerg. Med. Australas..

[2] Garakani A., Jaffe R.J., Savla D., Welch A.K., Protin C.A., Bryson E.O., McDowell D.M. (2016). Neurologic, Psychiatric, and Other Medical Manifestations of Nitrous Oxide Abuse: A Systematic Review of the Case Literature. Am. J. Addict..

[3] Paulus M.C., Wijnhoven A.M., Maessen G.C., Blankensteijn S.R., van der Heyden M.A.G. (2021). Does Vitamin B12 Deficiency Explain Psychiatric Symptoms in Recreational Nitrous Oxide Users? A Narrative Review. Clin. Toxicol..

[4] Lan S.Y., Kuo C.Y., Chou C.C., Kong S.S., Hung P.C., Tsai H.Y., Chen Y.C., Lin J.J., Chou I.J., Lin K.L. (2019). Recreational Nitrous Oxide Abuse Related Subacute Combined Degeneration of the Spinal Cord in Adolescents—A Case Series and Literature Review. Brain Dev..

[5] Bonev V., Wyatt M., Barton M.J., Leitch M.A. (2023). Severe Length-dependent Peripheral Polyneuropathy in a Patient with Subacute Combined Spinal Cord Degeneration Secondary to Recreational Nitrous Oxide Abuse: A Case Report and Literature Review. Clin. Case Rep..

[6] Sumnall H. (2022). Recreational Use of Nitrous Oxide. BMJ.

[7] World Health Organization (2019). World Health Organization Model List of Essential Medicines, 21st List.

[8] European Monitoring Centre for Drugs and Drug Abuse (2023). Recreational Use of Nitrous Oxide: A Growing Concern for Europe.

[9] Xiang Y., Li L., Ma X., Li S., Xue Y., Yan P., Chen M., Wu J. (2021). Recreational Nitrous Oxide Abuse: Prevalence, Neurotoxicity, and Treatment. Neurotox. Res..

[10] Evans E.B., Evans M.R. (2021). Nangs, Balloons, and Crackers. Recreational Nitrous Oxide Neurotoxicity. AJGP.

[11] Office for National Statistics (ONS) (2022). Drug Misuse in England and Wales: Year Ending June 2022.

[12] Chevaliercurt M.J., Grzych G., Tard C., Lannoy J., Deheul S., Hanafi R., Douillard C., Vamecq J., Grzych G., Tard C. (2022). Nitrous Oxide Abuse in the Emergency Practice, and Review of Toxicity Mechanisms and Potential Markers. Food Chem. Toxicol..

[13] Banjongjit A., Sutamnartpong P., Mahanupap P., Phanachet P., Thanakitcharu S. (2023). Nitrous Oxide-Induced Cerebral Venous Thrombosis: A Case Report, Potential Mechanisms, and Literature Review. Cureus.

[14] Sluyts Y., Vanherpe P., Amir R., Vanhoenacker F., Vermeersch P. (2022). Vitamin B12 Deficiency in the Setting of Nitrous Oxide Abuse: Diagnostic Challenges and Treatment Options in Patients Presenting with Subacute Neurological Complications. Acta Clin. Belg. Int. J. Clin. Lab. Med..

[15] Tani J., Weng H.Y., Chen H.J., Chang T.S., Sung J.Y., Lin C.S.Y. (2019). Elucidating Unique Axonal Dysfunction between Nitrous Oxide Abuse and Vitamin B12 Deficiency. Front. Neurol..

[16] Porruvecchio E., Shrestha S., Khuu B., Rana U.I., Zafar M., Zafar M., Kiani A., Hadid A. (2022). Functional Vitamin B12 Deficiency in Association With Nitrous Oxide Inhalation. Cureus.

[17] Maheshwari M., Athiraman H. (2022). Whippets Causing Vitamin B12 Deficiency. Cureus.

[18] Edigin E., Ajiboye O., Nathani A. (2019). Nitrous Oxide-Induced B12 Deficiency Presenting With Myeloneuropathy. Cureus.

[19] Strauss J., Qadri S.F. (2021). Myelopathy Secondary to Vitamin B12 Deficiency Induced by Nitrous Oxide Abuse. Cureus.

[20] Oulkadi S., Peters B., Vliegen A.S. (2022). Thromboembolic Complications of Recreational Nitrous Oxide (Ab)Use: A Systematic Review. J. Thromb. Thrombolysis.

[21] den Uil S.H., Vermeulen E.G.J., Metz R., Rijbroek A., de Vries M. (2018). Aortic Arch Thrombus Caused by Nitrous Oxide Abuse. J. Vasc. Surg. Cases Innov. Tech..

[22] Pratt D.N., Patterson K.C., Quin K. (2020). Venous Thrombosis after Nitrous Oxide Abuse, a Case Report. J. Thromb. Thrombolysis.

[23] Vollenbrock S.E., Fokkema T.M., Leijdekkers V.J., Vahl A.C., Konings R., van Nieuwenhuizen R.C. (2021). Nitrous Oxide Abuse Associated with Severe Thromboembolic Complications. Eur. J. Vasc. Endovasc. Surg..

[24] Fang X., Yu M., Zheng D., Gao H., Li W., Ma Y. (2023). Electrophysiologic Characteristics of Nitrous-Oxide-Associated Peripheral Neuropathy: A Retrospective Study of 76 Patients. J. Clin. Neurol..

[25] Dang X.T., Nguyen T.X., Nguyen T.T.H., Ha H.T. (2021). Nitrous Oxide-Induced Neuropathy among Recreational Users in Vietnam. Int. J. Environ. Res. Public Health.

[26] Wang Q., Duan X., Dong M., Sun S., Zhang P., Liu F., Wang L., Wang R. (2022). Clinical Feature and Sural Biopsy Study in Nitrous Oxide-Induced Peripheral Neuropathy. PLoS ONE.

[27] Vollhardt R., Mazoyer J., Bernardaud L., Haddad A., Jaubert P., Coman I., Manceau P., Mongin M., Degos B. (2022). Neurological Consequences of Recreational Nitrous Oxide Abuse during SARS-CoV-2 Pandemic. J. Neurol..

[28] Sood R., Parent T. (2022). Peripheral Polyneuropathy and Acute Psychosis from Chronic Nitrous Oxide Poisoning: A Case Report with Literature Review. Medicine.

[29] Dong X., Ba F., Wang R., Zheng D. (2019). Imaging Appearance of Myelopathy Secondary to Nitrous Oxide Abuse: A Case Report and Review of the Literature. Int. J. Neurosci..

[30] van Riel A.J.H.P., Hunault C.C., van den Hengel-Koot I.S., Nugteren-van Lonkhuyzen J.J., de Lange D.W., Hondebrink L. (2022). Alarming Increase in Poisonings from Recreational Nitrous Oxide Use after a Change in EU-Legislation, Inquiries to the Dutch Poisons Information Center. Int. J. Drug Policy.

[31] Duque M.A., Kresak J.L., Falchook A., Harris N.S. (2015). Nitrous Oxide Abuse and Vitamin B12 Action in a 20-Year-Old Woman: A Case Report. Lab. Med..

[32] Chiang T.T., Hung C.T., Wang W.M., Lee J.T., Yang F.C. (2013). Recreational Nitrous Oxide Abuse-Induced Vitamin B12 Deficiency in a Patient Presenting with Hyperpigmentation of the Skin. Case Rep. Dermatol..

[33] Razaq J., Qureshi S. (2020). Recreational Nitrous Oxide Abuse Causing B12 Deficiency with Subacute Combined Degeneration of the Spinal Cord: A Case Report. J. Fam. Med. Prim. Care.

[34] Li Y., Dong J., Xu R., Feng F., Kan W., Ding H., Wang X., Chen Y., Wang X., Zhu S. (2021). Clinical Epidemiological Characteristics of Nitrous Oxide Abusers: A Single-Center Experience in a Hospital in China. Brain Behav..

[35] Inquimbert C., Maitre Y., Moulis E., Gremillet V., Tramini P., Valcarcel J., Carayon D. (2022). Recreational Nitrous Oxide Use and Associated Factors among Health Profession Students in France. Int. J. Environ. Res. Public Health.

[36] Dufayet L., Caré W., Laborde-Casterot H., Chouachi L., Langrand J., Vodovar D. (2022). Possible Impact of the COVID-19 Pandemic on the Recreational Use of Nitrous Oxide in the Paris Area, France. Rev. Med. Interne.

[37] Cohen L., Duroy D., Perozziello A., Sasportes A., Lejoyeux M., Geoffroy P.A. (2023). A Cross-Sectional Study: Nitrous Oxide Abuse in Parisian Medical Students. Am. J. Addict..

[38] Ehirim E.M., Naughton D.P., Petróczi A. (2018). No Laughing Matter: Presence, Consumption Trends, Drug Awareness, and Perceptions of “Hippy Crack” (Nitrous Oxide) among Young Adults in England. Front. Psychiatry.

[39] von Elm E., Altman D.G., Egger M., Pocock S.J., Gøtsche P.C., Vandenbroucke J.P. (2007). STROBE Initiative Strengthening the Reporting of Observational Studies in Epidemiology (StroBE) Statement: Guidelines for Reporting Observational Studies. BMJ.

[40] Cecchini E., Schino S., Gambadoro N., Ricciardi L., Trio O., Biondi-Zoccai G., Sangiorgi G. (2022). Facing the Pandemic with a Smile: The Case of Memedical and Its Impact on Cardiovascular Professionals. Minerva Cardiol. Angiol..

[41] Riccò M., Zaniboni A., Satta E., Ranzieri S., Cerviere M.P., Marchesi F., Peruzzi S. (2022). West Nile Virus Infection: A Cross-Sectional Study on Italian Medical Professionals during Summer Season 2022. Trop. Med. Infect. Dis..

[42] Riccò M., Ferraro P., Camisa V., Satta E., Zaniboni A., Ranzieri S., Baldassarre A., Zaffina S., Marchesi F. (2022). When a Neglected Tropical Disease Goes Global: Knowledge, Attitudes and Practices of Italian Physicians towards Monkeypox, Preliminary Results. Trop. Med. Infect. Dis..

[43] Riccò M., Ferraro P., Camisa V., Di Palma P., Minutolo G., Ranzieri S., Zaffina S., Baldassarre A., Restivo V. (2022). Managing of Migraine in the Workplaces: Knowledge, Attitudes and Practices of Italian Occupational Physicians. Medicina.

[44] Riccò M., Gualerzi G., Ranzieri S., Ferraro P., Bragazzi N.L. (2020). Knowledge, Attitudes, Practices (KAP) of Italian Occupational Physicians towards Tick Borne Encephalitis. Trop. Med. Infect. Dis..

[45] Zingg A., Siegrist M. (2012). Measuring People’s Knowledge about Vaccination: Developing a One-Dimensional Scale. Vaccine.

[46] Betsch C., Wicker S. (2014). Personal Attitudes and Misconceptions, Not Official Recommendations Guide Occupational Physicians’ Vaccination Decisions. Vaccine.

[47] Rosenstock I.M. (1974). Historical Origins of the Health Belief Model. Health Educ. Monogr..

[48] Janz N.K., Becker M.H. (1984). The Health Belief Model: A Decade Later. Health Educ. Q..

[49] Carpenter C.J. (2010). A Meta-Analysis of the Effectiveness of Health Belief Model Variables in Predicting Behavior. Health Commun..

[50] Mo P.K.H., Lau J.T.F. (2015). Influenza Vaccination Uptake and Associated Factors among Elderly Population in Hong Kong: The Application of the Health Belief Model. Health Educ. Res..

[51] Imorde L., Möltner A., Runschke M., Weberschock T., Rüttermann S., Gerhardt-Szép S. (2020). Adaptation and Validation of the Berlin Questionnaire of Competence in Evidence-Based Dentistry for Dental Students: A Pilot Study. BMC Med. Educ..

[52] Riccò M., Ferraro P., Ranzieri S., Boldini G., Zanella I., Marchesi F. (2023). Legionnaires’ Disease in Occupational Settings: A Cross-Sectional Study from Northeastern Italy (2019). Trop. Med. Infect. Dis..

[53] Haddock G., Maio G.R., Hewstone M., Stroebe W., Jonas K. (2012). 6 Attitudes: Content, Structure and Functions. An Introduction to Social Psychology.

[54] Arghittu A., Dettori M., Azara A., Gentili D., Serra A., Contu B., Castiglia P. (2020). Flu Vaccination Attitudes, Behaviours, and Knowledge among Health Workers. Int. J. Environ. Res. Public Health.

[55] European Union, European Council, European Parliament (2016). Europea Union (EU) Regulation N. 2016/679 on the Protection of Natural Persons with Regard to the Processing of Personal Data and on the Free Movement of Such Data, and Repealing Directive 95/46/EC (General Data Protection Regulation).

[56] Perino J., Tournier M., Mathieu C., Letinier L., Peyré A., Perret G., Pereira E., Fourrier-Réglat A., Pollet C., Fatseas M. (2022). Psychoactive Substance Use among Students: A Cross-Sectional Analysis. Fundam. Clin. Pharmacol..

[57] Roberts D., Farahmand P., Wolkin A. (2020). Nitrous Oxide Inhalant Use Disorder Preceding Symptoms Concerning for Primary Psychotic Illness. Am. J. Addict..

[58] Hew A., Lai E., Radford E. (2018). Nitrous Oxide Abuse Presenting with Acute Psychosis and Peripheral Neuropathy. Aust. N. Z. J. Psychiatry.

[59] Matsuda N., Wakakuri H., Uehara K., Hyodo H., Ohara T., Yasutake M. (2022). A Case of Fever, Impaired Consciousness, and Psychosis Caused by Nitrous Oxide Abuse and Misdiagnosed as Acute Meningitis. J. Nippon Med. Sch..

[60] Wu G., Wang S., Wang T., Han J., Yu A., Feng C., Wang Y., Liu S. (2022). Neurological and Psychological Characteristics of Young Nitrous Oxide Abusers and Its Underlying Causes During the COVID-19 Lockdown. Front. Public Health.

[61] Kaar S.J., Ferris J., Waldron J., Devaney M., Ramsey J., Winstock A.R. (2016). Up: The Rise of Nitrous Oxide Abuse. An International Survey of Contemporary Nitrous Oxide Use. J. Psychopharmacol..

[62] Huizink A.C. (2022). Trends and Associated Risks in Adolescent Substance Use: E-Cigarette Use and Nitrous Oxide Use. Curr. Opin. Psychol..

[63] Lewis B., Nelson G., Vu T., Judge B. (2021). No Laughing Matter—Myeloneuropathy Due to Heavy Chronic Nitrous Oxide Abuse. Am. J. Emerg. Med..

[64] Sun W., Liao J.P., Hu Y., Zhang W., Ma J., Wang G.F. (2019). Pulmonary Embolism and Deep Vein Thrombosis Caused by Nitrous Oxide Abuse: A Case Report. World J. Clin. Cases.

[65] Liu M., Zhang J., Bu B. (2020). Isolated Cortical Vein Thrombosis after Nitrous Oxide Use in a Young Woman: A Case Report. BMC Neurol..

[66] Peng C., Liu X., Wu K., Lang H., He L., Chen N. (2023). Nitrous Oxide Inhalation-Induced Cerebral Venous Sinus Thrombosis in a 20-Year-Old Man: A Case Report. Heliyon.

[67] Pedersen O.B., Hvas A.M., Grove E.L. (2021). A 19-Year-Old Man with a History of Recreational Inhalation of Nitrous Oxide with Severe Peripheral Neuropathy and Central Pulmonary Embolism. Am. J. Case Rep..

[68] Molina M.F., Al Saud A.A., Al Mulhim A.A., Liteplo A.S., Shokoohi H. (2020). Nitrous Oxide Inhalant Abuse and Massive Pulmonary Embolism in COVID-19. Am. J. Emerg. Med..

[69] Parein G., Bollens B. (2023). Nitrous Oxide-Induced Polyneuropathy, Pancytopenia and Pulmonary Embolism: A Case Report. J. Med. Case Rep..

[70] de Valck L., Defelippe V.M., Bouwman N.A.M.G. (2021). Cerebral Venous Sinus Thrombosis: A Complication of Nitrous Oxide Abuse. BMJ Case Rep..

[71] Farhat W., Pariente A., Mijahed R. (2022). Extensive Cerebral Venous Thrombosis Secondary to Recreational Nitrous Oxide Abuse. Cerebrovasc. Dis..

[72] Lin S.-S., Fan I.-W., Chen C.-Y., Su Y.-J. (2022). A Nitrous Oxide Abuser Presenting with Cerebral Venous Thrombosis: A Case Report. Med. Int..

[73] Anderson D., Beecher G., Van DIjk R., Hussain M., Siddiqi Z., Ba F. (2018). Subacute Combined Degeneration from Nitrous Oxide Abuse in a Patient with Pernicious Anemia. Can. J. Neurol. Sci..

[74] Einsiedler M., Voulleminot P., Demuth S., Kalaaji P., Bogdan T., Gauer L., Reschwein C., Nadaj-Pakleza A., de Sèze J., Kremer L. (2022). A Rise in Cases of Nitrous Oxide Abuse: Neurological Complications and Biological Findings. J. Neurol..

[75] Kingma T.J., Bascoy S., Altaf M.D., Surampudy A., Chaudhry B. (2022). Subacute Combined Degeneration of the Spinal Cord: A Consequence of Recreational Nitrous Oxide Use. Cureus.

[76] McArdle D.J.T., Gaillard F. (2020). Pernicious Azotaemia? A Case Series of Subacute Combined Degeneration of the Cord Secondary to Nitrous Oxide Abuse. J. Clin. Neurosci..

[77] Bajaj D., Agrawal A., Gupta S., Bajaj S. (2018). Recreational Nitrous Oxide Abuse Causing Ischemic Stroke in a Young Patient: A Rare Case Report. Cureus.

[78] Berling E., Fargeot G., Aure K., Tran T.H., Kubis N., Lozeron P., Zanin A. (2022). Nitrous Oxide-Induced Predominantly Motor Neuropathies: A Follow-up Study. J. Neurol..

[79] Fang X., Li W., Gao H., Ma Y., Dong X., Zheng D. (2020). Skin Hyperpigmentation: A Rare Presenting Symptom of Nitrous Oxide Abuse. Clin. Toxicol..

[80] Yu M., Qiao Y., Li W., Fang X., Gao H., Zheng D., Ma Y. (2022). Analysis of Clinical Characteristics and Prognostic Factors in 110 Patients with Nitrous Oxide Abuse. Brain Behav..

[81] Temple C., Zane Horowitz B. (2022). Nitrous Oxide Abuse Induced Subacute Combined Degeneration despite Patient Initiated B12 Supplementation. Clin. Toxicol..

[82] Patwardhan M.B., Samsa G.P., Lipton R.B., Matchar D.B. (2006). Changing Physician Knowledge, Attitudes, and Beliefs about Migraine: Evaluation of a New Educational Intervention. Headache.

[83] Di Prinzio R.R., Nigri A.G., Zaffina S. (2021). Total Worker Heath Strategies in Italy: New Challenges and Opportunities for Occupational Health and Safety Practice. J. Health Soc. Sci..

[84] Remoundou K., Brennan M., Sacchettini G., Panzone L., Butler-Ellis M.C., Capri E., Charistou A., Chaideftou E., Gerritsen-Ebben M.G., Machera K. (2015). Perceptions of Pesticides Exposure Risks by Operators, Workers, Residents and Bystanders in Greece, Italy and the UK. Sci. Total Environ..

[85] Riccò M., Ferraro P., Peruzzi S., Balzarini F., Ranzieri S. (2021). Mandate or Not Mandate: Knowledge, Attitudes, and Practices of Italian Occupational Physicians towards SARS-CoV-2 Immunization at the Beginning of Vaccination Campaign. Vaccines.

[86] Giaccardi M., Anselmino M., Del Greco M., Mascia G., Paoletti Perini A., Mascia P., De Ferrari G.M., Picano E. (2021). Radiation Awareness in an Italian Multispecialist Sample Assessed with a Web-Based Survey. Acta Cardiol..

[87] Heiervang E., Goodman R. (2011). Advantages and Limitations of Web-Based Surveys: Evidence from a Child Mental Health Survey. Soc. Psychiat. Epidemiol..

[88] Noale M., Trevisan C., Maggi S., Incalzi R.A., Pedone C., Di Bari M., Adorni F., Jesuthasan N., Sojic A., Galli M. (2020). The Association between Influenza and Pneumococcal Vaccinations and SARS-CoV-2 Infection: Data from the EPICOVID19 Web-Based Survey. Vaccines.

[89] Riccò M., Cattani S., Casagranda F., Gualerzi G., Signorelli C. (2017). Knowledge, Attitudes, Beliefs and Practices of Occupational Physicians towards Vaccinations of Health Care Workers: A Cross Sectional Pilot Study in North-Eastern Italy. Int. J. Occup. Med. Environ. Health.

[90] Riccò M., Zaniboni A., Satta E., Baldassarre A., Cerviere M.P., Marchesi F., Peruzzi S. (2022). Management and Prevention of Traveler’s Diarrhea: A Cross-Sectional Study on Knowledge, Attitudes, and Practices in Italian Occupational Physicians (2019 and 2022). Trop. Med. Infect. Dis..

[91] Trumello C., Bramanti S.M., Ballarotto G., Candelori C., Cerniglia L., Cimino S., Crudele M., Lombardi L., Pignataro S., Viceconti M.L. (2020). Psychological Adjustment of Healthcare Workers in Italy during the COVID-19 Pandemic: Differences in Stress, Anxiety, Depression, Burnout, Secondary Trauma, and Compassion Satisfaction between Frontline and Non-Frontline Professionals. Int. J. Environ. Res. Public Health.

[92] Vicarelli G., Pavolini E. (2015). Health Workforce Governance in Italy. Health Policy.

[93] Riccò M., Vezzosi L., Balzarini F. (2020). Challenges Faced by the Italian Medical Workforce. Lancet.

[94] Miller N., Saunders I. (2007). Current Perceptions of Travelers’ Diarrhea Treatments and Vaccines: Results from a Postal Questionnaire Survey and Physician Interviews. J. Travel Med..

[95] Wilcox C.R., Calvert A., Metz J., Kilich E., Macleod R., Beadon K., Heath P.T., Khalil A., Finn A., Snape M.D. (2019). Attitudes of Pregnant Women and Healthcare Professionals Toward Clinical Trials and Routine Implementation of Antenatal Vaccination Against Respiratory Syncytial Virus: A Multicenter Questionnaire Study. Pediatr. Infect. Dis. J..

[96] Sutton S. (2000). Interpreting Cross-Sectional Data on Stages of Change. Psychol. Health.

[97] Mihara S., Higuchi S. (2017). Cross-Sectional and Longitudinal Epidemiological Studies of Internet Gaming Disorder: A Systematic Review of the Literature. Psychiatry Clin. Neurosci..

[98] Tostrud L., Thelen J., Palatnik A. (2022). Models of Determinants of COVID-19 Vaccine Hesitancy in Non-Pregnant and Pregnant Population: Review of Current Literature. Hum. Vaccin. Immunother..

[99] Betsch C., Schmid P., Heinemeier D., Korn L., Holtmann C., Böhm R. (2018). Beyond Confidence: Development of a Measure Assessing the 5C Psychological Antecedents of Vaccination. PLoS ONE.

[100] Riccò M., Peruzzi S. (2022). Tetanus Vaccination Status and Vaccine Hesitancy in Amateur Basketball Players (Italy, 2020). Vaccines.

[101] Prochaska J.O., Velicer W.F. (1997). The Transtheoretical Model of Health Behavior Change. Am. J. Health Promot..

[102] Lipschitz J.M., Fernandez A.C., Elsa Larson H., Blaney C.L., Meier K.S., Redding C.A., Prochaska J.O., Paiva A.L. (2013). Validation of Decisional Balance and Self-Efficacy Measures for HPV Vaccination in College Women. Am. J. Health Promot..

[103] Arnett D.K., Claas S.A. (2017). Introduction to Epidemiology. Clinical and Translational Science: Principles of Human Research.

[104] di Girolamo N., Mans C. (2018). Research Study Design. Miller—Fowler’s Zoo and Wild Animal Medicine Current Therapy.

[105] Betsch C., Wicker S. (2012). E-Health Use, Vaccination Knowledge and Perception of Own Risk: Drivers of Vaccination Uptake in Medical Students. Vaccine.

[106] Kow R.Y., Mohamad Rafiai N., Ahmad Alwi A.A., Low C.L., Ahmad M.W., Zakaria Z., Zulkifly A.H. (2022). COVID-19 Infodemiology: Association Between Google Search and Vaccination in Malaysian Population. Cureus.

[107] Bragazzi N.L. (2013). Infodemiology and Infoveillance of Multiple Sclerosis in Italy. Mult. Scler. Int..

[108] Ciaffi J., Meliconi R., Landini M.P., Mancarella L., Brusi V., Faldini C., Ursini F. (2021). Seasonality of Back Pain in Italy: An Infodemiology Study. Int. J. Environ. Res. Public Health.

[109] Riccò M., Baldassarre A., Provenzano S., Corrado S., Cerviere M.P., Parisi S., Marchesi F., Bottazzoli M. (2022). Infodemiology of RSV in Italy (2017–2022): An Alternative Option for the Surveillance of Incident Cases in Pediatric Age?. Children.

[110] Riccò M., Valente M., Marchesi F. (2022). Are Symptoms Associated with SARS-CoV-2 Infections Evolving over Time?. Infect. Dis. Now.

[111] Cervellin G., Comelli I., Lippi G. (2017). Is Google Trends a Reliable Tool for Digital Epidemiology? Insights from Different Clinical Settings. J. Epidemiol. Glob. Health.

[112] Rovetta A. (2021). Reliability of Google Trends: Analysis of the Limits and Potential of Web Infoveillance During COVID-19 Pandemic and for Future Research. Front. Res. Metr. Anal..

[113] Grzych G., Deheul S., Gernez E., Davion J.B., Dobbelaere D., Carton L., Kim I., Guichard J.C., Girot M., Humbert L. (2023). Comparison of Biomarker for Diagnosis of Nitrous Oxide Abuse: Challenge of Cobalamin Metabolic Parameters, a Retrospective Study. J. Neurol..

[114] Ayre C., Scally A.J. (2014). Critical Values for Lawshe’s Content Validity Ratio: Revisiting the Original Methods of Calculation. Meas. Eval. Couns. Dev..

